# Histoplasma capsulatum var. duboisii: A Case of Histoplasmosis 50 Years After the Exposure

**DOI:** 10.7759/cureus.77854

**Published:** 2025-01-22

**Authors:** Ana Venâncio de Barros, Fátima Sousa Gonçalves, Luísa Mendonça, Dinah Carvalho, José Melo Cristino

**Affiliations:** 1 Clinical Pathology, Unidade Local de Saúde Santa Maria, Lisbon, PRT; 2 Infectious Disease, Unidade Local de Saúde Santa Maria, Lisbon, PRT

**Keywords:** africa, europe, histoplasma capsulatum var. dubossii, histoplasmosis, immunocompetent, laboratory approach, latente histoplasmosis

## Abstract

Histoplasmosis is a mycotic infection caused by *Histoplasma capsulatum*, a thermally dimorphic organism that presents two pathological variants in humans: *H. capsulatum* var. *capsulatum* and *H. capsulatum* var. *duboisii*. The latter is restricted to Africa, where both variants may coexist. *H. capsulatum* var. *duboisii *mainly affects the skin, subcutaneous tissues, and bone, with rare dissemination. Infections can exhibit prolonged latency, reactivating decades after exposure. This report describes a rare case of histoplasmosis due to *H. capsulatum *var.* duboisii*, including its laboratory approaches, in a 73-year-old immunocompetent male patient from Portugal who served in Angola in the 1970s. The patient presented with an exophytic gingival lesion that progressed over three months and a lesion on the left wrist joint. This case emphasizes the vital role of microbiological approaches in diagnosing histoplasmosis and highlights the need to consider it in individuals who have been in endemic regions, regardless of the temporal gap since exposure.

## Introduction

Histoplasmosis is a mycotic infection that can affect both immunocompetent and immunocompromised individuals. It is caused by the fungus *Histoplasma capsulatum*, which exhibits thermal dimorphism and presents two pathological variants in humans: *H. capsulatum* var. *capsulatum* and *H.* *capsulatum* var. *duboisii* [1,2]. The former variant is endemic in North and South America, while the latter is primarily found in Africa, mainly in Central and West Africa and Madagascar Island, although both variants can coexist on the African continent [1,2]. Cases of *H. capsulatum* var. *duboisii* are rare in Europe and all documented cases have been imported from Africa [2].

*H. capsulatum* is typically found in soils enriched with bird and bat excrement. The exact mode of transmission for *H. capsulatum* var. *duboisii* is not well established [1,2]; however, most cases have been associated with direct transmission following trauma [1]. While this variant predominantly affects the skin, subcutaneous tissues, lymph nodes, and bone [1,2], disseminated disease is rare and more likely to occur in individuals who are infected with human immunodeficiency virus (HIV) [1]. Infections caused by this species may exhibit a prolonged incubation period, with cases documented many years after the initial exposure [1].

The main morphological difference between the two variants lies in yeast cell size: *H. capsulatum* var. *capsulatum* exhibits smaller yeast cells, ranging from 2 to 5 μm, whereas *H. capsulatum* var. *duboisii* is characterized by larger yeast cells, ranging from 8 to 15 μm [1]. The laboratory diagnosis of histoplasmosis relies on a variety of methods, which include microscopy, culture, antigen and antibody detection, and molecular methods [3].

The microbiological culture of biological samples, demonstrating thermal dimorphism, remains the gold standard for confirming *Histoplasma spp.* infection [1,4]. Serum or urinary antigen detection provides a rapid, non-invasive, and highly sensitive method, particularly for disseminated cases and monitoring treatment [4,5]. Serologic testing through antibody detection is useful for chronic disease but has limitations, including false positives and lower sensitivity for *H. capsulatum* var. *duboisii* [1]. Molecular methods, such as polymerase chain reaction (PCR) for *Histoplasma* spp. DNA detection, are not yet standardized or recommended for the definitive diagnosis of histoplasmosis, according to the Infectious Disease Society of America (IDSA) [1,4,6]. The definitive diagnosis of histoplasmosis continues to rely on microbiological culture, which involves the isolation and identification of *H. capsulatum* from biological samples, or through histopathological confirmation [4,6,7].

Treatment options for histoplasmosis remain limited. In cases of severe disease, the recommended treatment is a combination of amphotericin B and itraconazole [8].

## Case presentation

**Figure 1:**
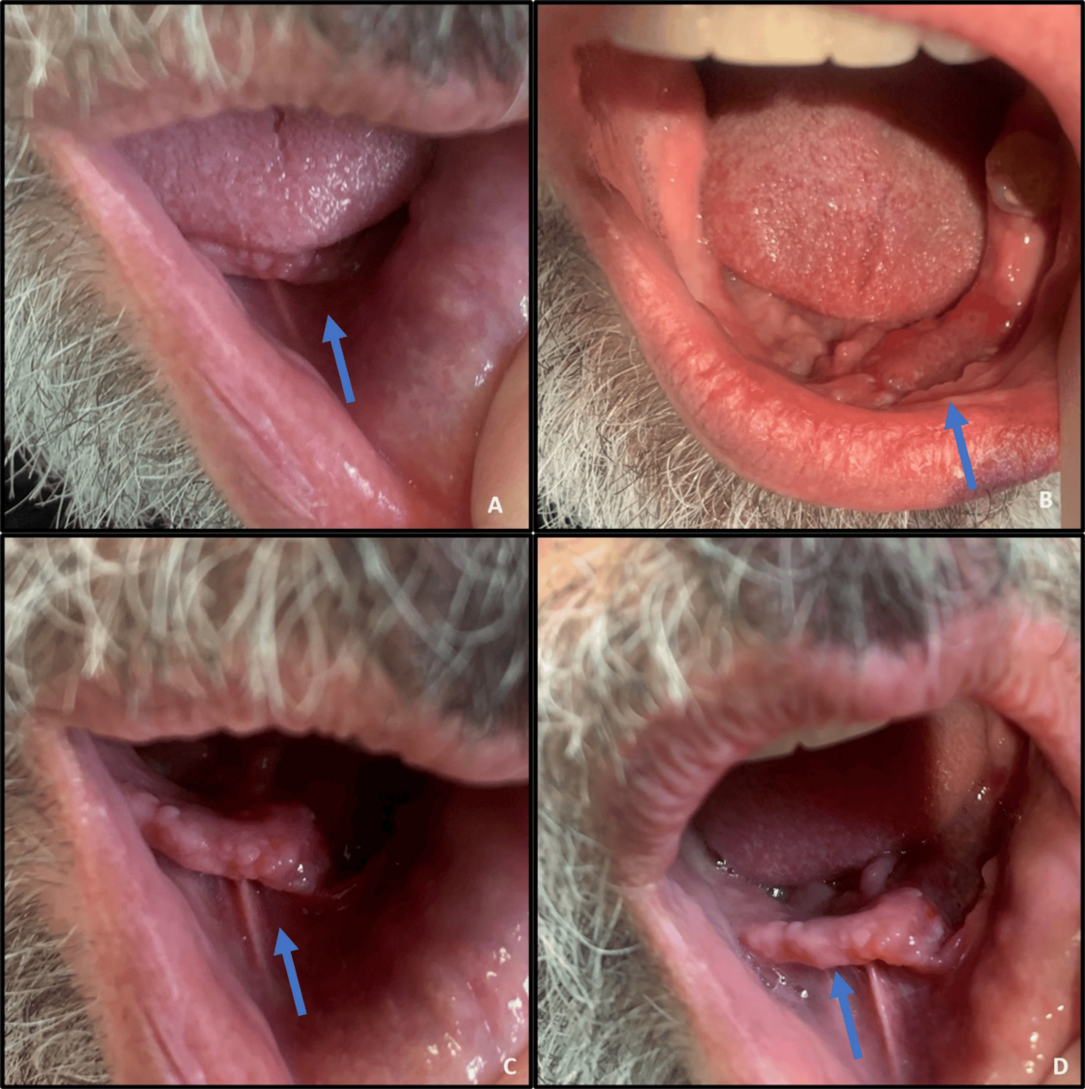
Exophytic gingival lesion located in the third quadrant Images show the same lesion from different angles and different magnifications.

A 73-year-old immunocompetent male patient from Portugal, who served as a soldier in Angola during the Portuguese Colonial War in the 1970s, was observed by a stomatology specialist due to an exophytic gingival lesion located in the third quadrant, measuring 3 cm, with three months of evolution (Figure 1). The lesion was subjected to biopsy and subsequently underwent histopathological and microbiological analysis.

**Figure 2:**
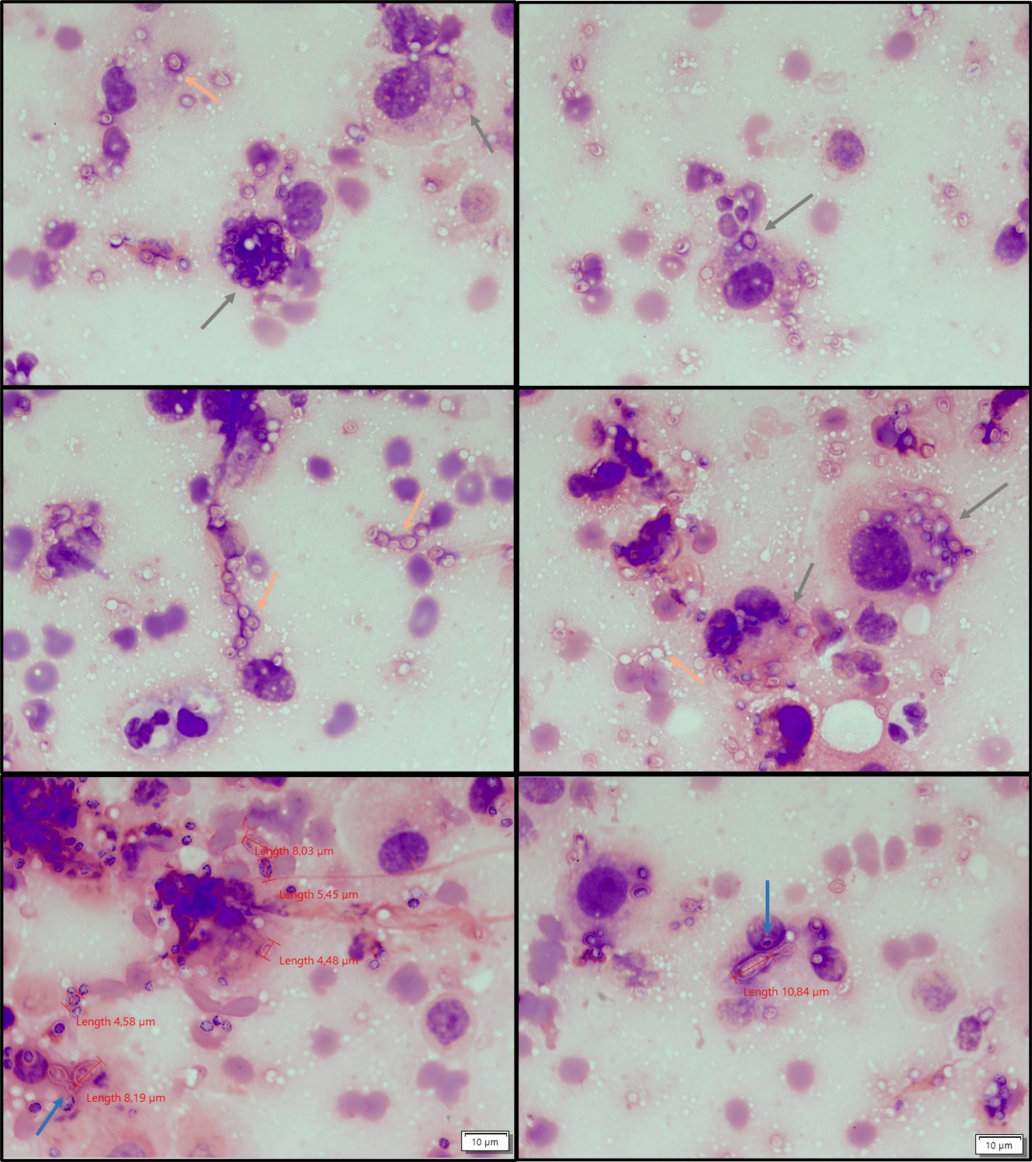
Direct Gram stain smear from the biopsy showing large yeast cells, some inside the macrophages (grey arrow), measuring 4 -11 µm, with narrow-based single budding (blue arrow). Other yeast cells spread through the smear (Orange arrow) Gram stain smear, 1000x magnification

The histopathological analysis identified histiocytes containing yeast cells morphologically consistent with *H. capsulatum*. The microbiological examination of a direct Gram stain smear from the biopsy revealed large yeast cells (4-11 µm) with narrow-based single budding (Figure 2), suggestive of *H. capsulatum*.

Upon this result, it was imperative to demonstrate the characteristic thermal dimorphism of *Histoplasma* spp. by performing two subcultures incubated at specific temperatures. Subcultures were prepared on blood agar and Sabouraud dextrose agar supplemented with gentamicin and chloramphenicol and were incubated at 25-30ºC in a 5% CO2 aerobic atmosphere. A second subculture on blood agar was incubated at 35-37ºC in a 5% CO2 aerobic atmosphere.

**Figure 3:**
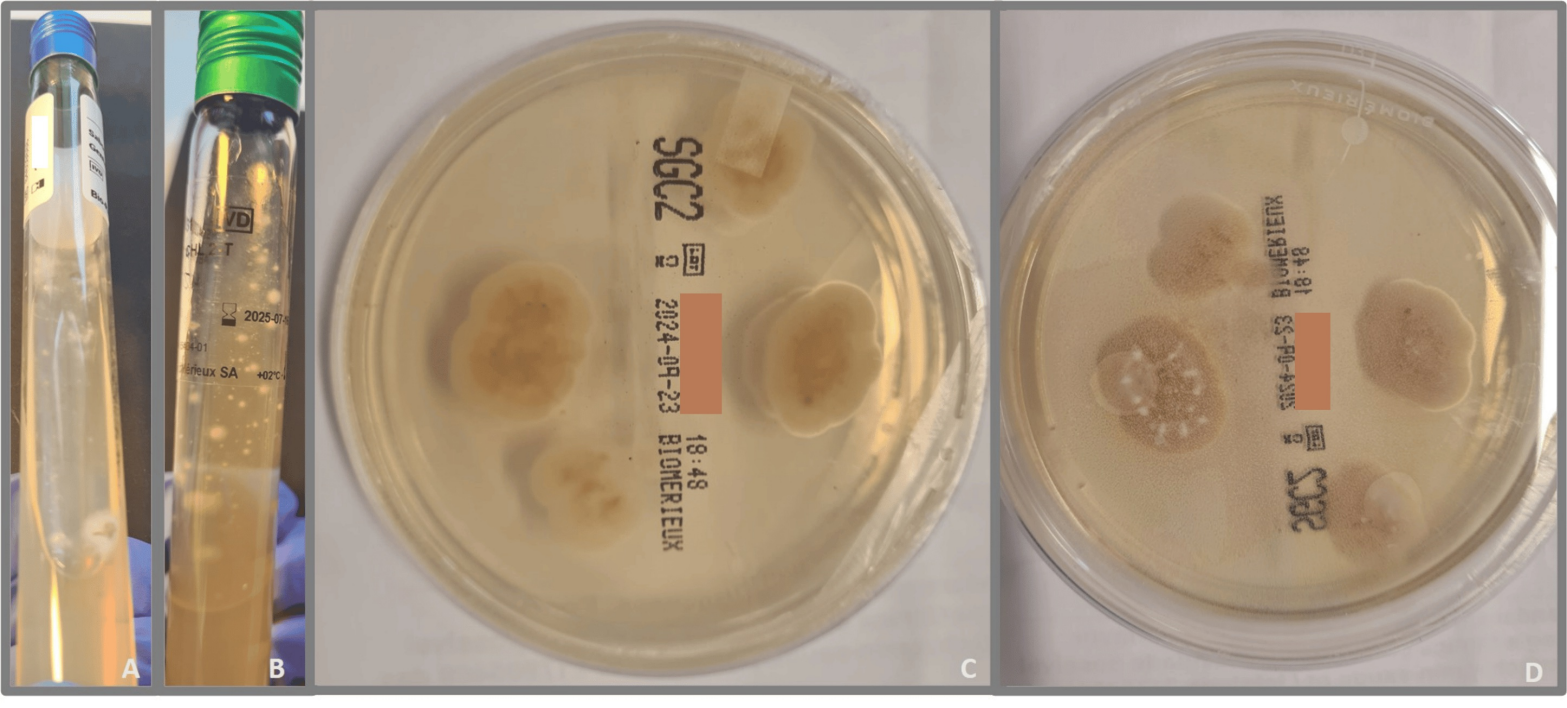
Sabouraud dextrose agar subcultures incubated at 25-30ºC showing granular, cottony white colonies on the 16th day of incubation Granular, cottony white colonies growing on Sabouraud dextrose agar (A) with cycloheximide on a slant tube, (B) on a slant tube, (C) on a plate (back), and (D) on a plate (front)

**Figure 4:**
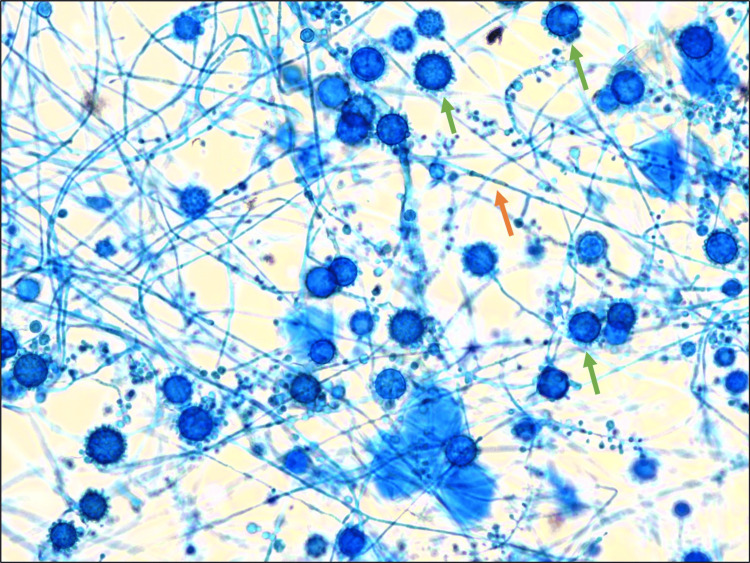
Direct microscopy with lactophenol blue from the colonies on the subcultures incubated at 25-30ºC showing fine septate hyphae (orange arrow) with large, round, thick-walled and tuberculate macroconidia (green arrow), characteristic of Histoplasma capsulatum Lactophenol blue, 400x magnification

On the 16th day of incubation, granular, cottony white colonies (Figure 3) were observed on the Sabouraud dextrose agar subcultures incubated at 25-30ºC. Direct microscopy with lactophenol blue of these colonies showed fine septate hyphae with large, round, thick-walled, and tuberculate macroconidia, characteristic of *H.* *capsulatum* (Figure 4). The microscopic appearance of mycelial form is the same for all variants of this fungus.

**Figure 5:**
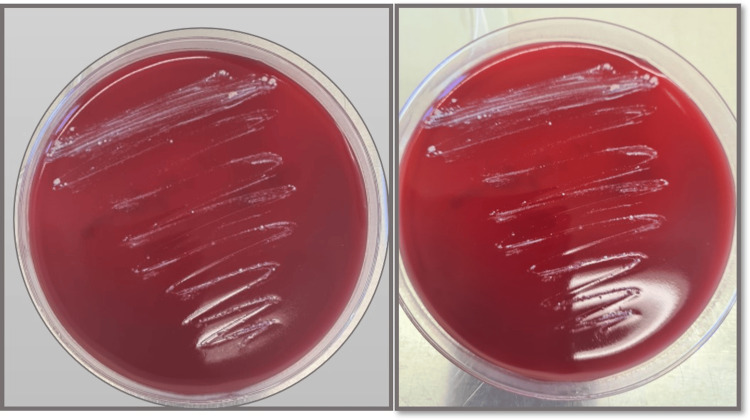
Blood agar incubated at 35-37ºC showing smooth, creamy and white yeast-like colonies on the 19th day of incubation

**Figure 6:**
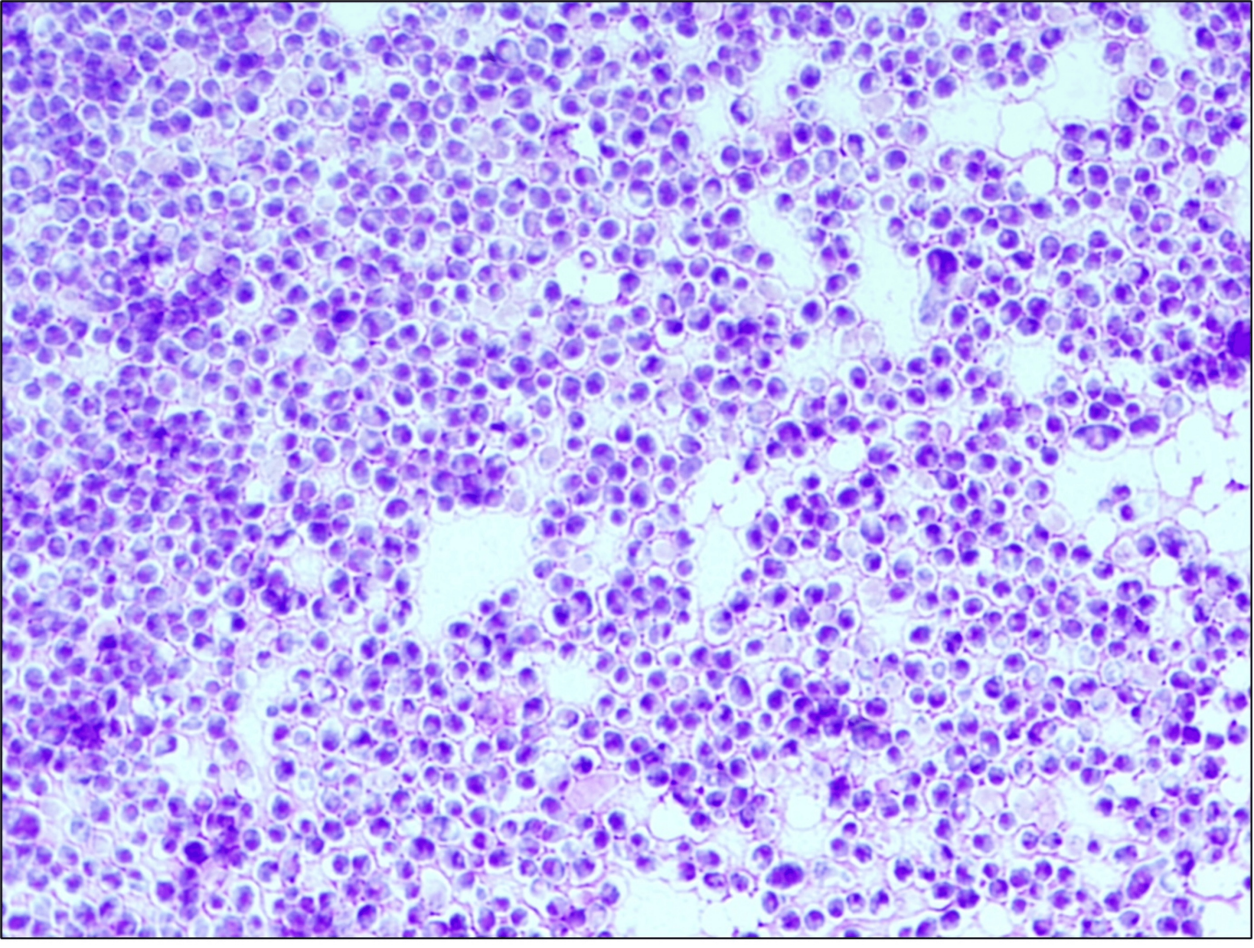
Giemsa-stained smear from yeast-like colonies on the blood agar showing large yeasts cells Giemsa-stained smear, 1000x magnification

On the other hand, on the blood agar subculture incubated at 35-37ºC, smooth, creamy, and white yeast-like colonies were observed on day 19 of incubation (Figure 5); a Giemsa-stained smear performed of these colonies showed large yeast cells (Figure 6), proving the thermal dimorphism of *Histoplasma* spp. The strain was subsequently sent to sequencing, which revealed a homology of 99.8% for *H. capsulatum* var. *duboisii*.

Following this outcome, the patient was subsequently referred for an infectious disease appointment, during which serological tests for HIV, hepatitis B, and hepatitis C infections were conducted, all revealed to be negative. Additionally, a lesion on the left wrist joint was identified. This lesion was biopsied and subjected to histopathological and microbiological analyses, yielding consistent results. In this patient, pulmonary involvement was also investigated, but the cultures of the respiratory samples were always negative.

The patient was diagnosed with chronic disseminated histoplasmosis involving two noncontiguous sites: an exophytic gingival lesion and a left wrist joint lesion. He was placed on an oral antifungal treatment regimen with itraconazole at a loading dose of 200 mg three times daily for three days, followed by a maintenance dose of 200 mg twice daily. Although the therapy was well tolerated for one month, resulting in a significant reduction of inflammatory signs in the left wrist, the patient later experienced a severe episode of rhabdomyolysis, which was attributed to a drug interaction between itraconazole and simvastatin. Following this complication, the patient was hospitalized, and the treatment was switched to liposomal amphotericin B for 14 days. After completing the course of amphotericin B, itraconazole was reintroduced at the same dosage, resulting in a favorable clinical outcome. The patient is currently continuing treatment with itraconazole; with complete resolution of the wrist lesion and significant improvement in the gingival lesion.

## Discussion

The diagnosis of histoplasmosis in a non-endemic region requires a high level of clinical suspicion. To consider this condition it is essential to conduct an epidemiological inquiry about any prior travel or residence in geographically endemic areas, regardless of the timeline of such exposure.

We presented a case involving a 73-year-old immunocompetent male patient who exhibited a latent form of histoplasmosis caused by *H. capsulatum* var. *duboisii*. This condition was reactivated following a latency period of 50 years, manifesting as chronic disseminated histoplasmosis that affected two noncontiguous body sites. *H. capsulatum* var. *duboisii* has predominantly been documented in individuals who are not infected with HIV [8], as it happened in this patient. It is possible that a long-lasting local infection, occurring in the presence of a competent immune system prevented dissemination. *H. capsulatum* var. *duboisii* can develop a state of latency within granulomas with local reactivation several years after initial infection. Thus, the time between infection and reactivation was long, decades, as reported in several other cases in the literature [8]. 

It is plausible that age-related immunosuppression played a role in the subsequent manifestation of the disease. While the disseminated infection is usually acute, chronic disseminated infection has also been described and is generally observed in older adults who do not exhibit any apparent immunosuppression [9].

The diagnosis of histoplasmosis poses clinical and laboratory challenges. This case highlights the need for specialized laboratory skills to accurately identify and differentiate this fungus from other saprophytes and dimorphic fungi.

The microbiological analysis was conducted in accordance with the histopathological suspicion from the gingival biopsy, with the diagnosis confirmed through direct examination and culture, the latter remaining the reference method. Although microbiological culture remains the gold standard for diagnosis, its sensitivity is influenced by both the duration of the incubation period and the type of culture media used [1]. It is recommended that the incubation period be no less than three weeks, using Sabourauddextrose agar in combination with other nutrient-rich media, such as blood agar or chocolate agar [1]. In this case, the fungus was successfully isolated on the 16th day of incubation (see Figure 3), and its identity was confirmed by demonstrating thermal dimorphism on the 19th day (see Figure 5). Further characterization of the specific variant was identified through molecular sequencing.

The two variants of *H. capsulatum* are indistinguishable by culture; however, it is possible to observe certain morphological differences, particularly in the yeast cell size, when examining the infective tissue sample through direct microscopy. The yeast cells observed in the direct microscopy of the sample exhibited a slightly smaller size range (4-11 µm) than that described in the literature (8-15 µm).

When investigating a potential case of histoplasmosis in the microbiology laboratory, it is important to differentiate it from other morphologically similar fungi. Notably, *Sepedonium* spp. is a non-pathogenic saprophyte that exhibits morphological similarities to the filamentous phase of *H. capsulatum*, as well as to the yeast cells of *Blastomyces dermatitidis*, a thermal dimorphic fungus endemic to North America but not in Africa. We successfully excluded this saprophytic fungus by using Sabouraud dextrose agar containing cycloheximide (see Figure 3A), which inhibits their growth. In this instance, colonies were observed growing on this agar. Furthermore, by demonstrating thermal dimorphism, we were able to exclude the possibility of a saprophytic fungus. The theory of *B. dermatitidis* was dismissed due to the unlikely clinical history, the morphology of the yeast cells, which exhibited single narrow budding and a size range inconsistent with *B. dermatitidis*, and the results of molecular sequencing.

Other methods such as urinary antigen detection and serological antibody testing could have been helpful in achieving a faster diagnosis; however, in this patient, no clinical suspicion was raised until the histopathological and microbiological results became available.

The management of histoplasmosis caused by *H. capsulatum* var. *duboisii* is not well defined and lacks specific treatment guidelines. Consequently, patients diagnosed with this condition are often treated according to the established protocols for histoplasmosis caused by *H. capsulatum* var. *capsulatum*. The preferred therapeutic agents for treating histoplasmosis include liposomal amphotericin B and itraconazole [8]. In the case presented, the patient received both medications throughout the illness, without conducting a pharmacokinetic study, but with a favorable clinical outcome.

In the case under consideration, the disease was most likely due to the reactivation of a previously acquired infection that occurred during military deployment in Angola 50 years ago. Both the reactivation process and the clinical manifestations of the disease may have been influenced by age-related immunosuppression.

## Conclusions

This report discusses a rare disease observed in Europe and highlights the importance of direct microscopy of samples for timely preliminary reports, guiding mycological procedures and determining appropriate incubation times, ultimately leading to the diagnosis of histoplasmosis. Furthermore, it emphasizes the need to consider histoplasmosis in individuals with a history of exposure, regardless of the timeline of its occurrence.
